# The Concept of a Large Group-Based Approach for Intensive Smoking Cessation: The Gold Standard Program (GSP)

**DOI:** 10.3390/ijerph21111423

**Published:** 2024-10-26

**Authors:** Mie Sylow Liljendahl, Marianne Ahrenkiel Søgaard, Hanne Tønnesen

**Affiliations:** 1 Clinical Health Promotion Centre, WHO-CC, Parker Institute, Bispebjerg & Frederiksberg Hospital, 2000 Frederiksberg, Denmark; hanne.tonnesen@regionh.dk; 2 Development and Prevention, Silkeborg Kommune, 8600 Silkeborg, Denmark

**Keywords:** gold standard program, intensive smoking cessation intervention, large group, national database

## Abstract

In this study, the effect of the intensive “Quitter” intervention for smoking cessation was examined and compared to the standardized intensive intervention used in Denmark. The Quitter intervention, based on the Gold Standard Program (Q-GSP), involves large groups of approximately 70 participants, while the standardized GSP typically involves groups of 10–15 participants. In total, 105 and 14,289 smokers participated in the Q-GSP and the standardized GSP, respectively, between 2022 and 2023. Data from the Danish STOPbase database were used, with validated information from one municipality for the Q-GSP. Successful smoking cessation was defined as abstinence from smoking at the end of the intervention and continuous abstinence at the 6-month follow-up. The results showed that 73% of the participants in the Quitter group successfully stopped smoking at the end of the interventions, compared to 50% in the standardized I-SCI group. At the 6-month follow-ups, 49% of the Quitter intervention group members maintained abstinence versus 31% of the standardized I-SCI group, with a statistically significant relative risk (RR) of 2.18 (95% CI 1.48–3.22). Compliance and satisfaction were high in both groups. These findings indicate the potential for large group-based interventions to be broadly integrated into public health strategies.

## 1. Introduction

Smoking cessation interventions in groups remain a common format, gaining popularity and becoming a well-established approach. These interventions often include identifying the motives for the group members’ behavior, providing opportunities for social learning, generating emotional experiences and self-control strategies, and imparting information and skills in relation to smoking cessation [1,2]. A specific therapeutic benefit of the group format is the opportunity it provides for individuals to share problems and experiences with others who are also attempting to stop smoking [1]. Group-based interventions have been shown to be more effective than usual care, self-help, and less intensive interventions [1,3]. However, a Cochrane Review reported that the average cessation rates associated with group-based treatments ranged from 9% to 20%. Clearly, there is a need for more effective group-based approaches, as well as alternative options to these settings that might offer equal or greater effects [1].

The Gold Standard Program (GSP) has been implemented in Denmark since 1995. This program is widely used in primary healthcare and represents the majority of the smoking cessation interventions offered. The intervention is a comprehensive approach that includes five to six meetings over six weeks. The therapists facilitating the program often have a health professional background with additional training to become certified and are equipped to deliver manual-based patient education. The program is free of charge and includes access to free nicotine replacement therapy. The GSP has demonstrated a high efficacy in randomized trials as well as a high effectiveness and cost-effectiveness in real-life settings [4,5,6,7]. A comprehensive review showed that intensive smoking cessation interventions, like this program, have doubled the success rate compared to briefer interventions, including motivational intervention techniques [8].

Effective smoking cessation intervention is crucial as a means to prevent smoking-related morbidity and mortality [9]. However, smoking cessation methods often face recruitment challenges. Despite progress in reducing smoking prevalence [10], the challenges in recruitment are recurrent and only a small proportion of smokers receive appropriate intervention, highlighting the need for innovative approaches to improve or extend the existing strategies. The intensive smoking cessation intervention, called “Quitter”, uses the GSP with a new recruiting process. Unlike the standardized GSP, which typically involves groups of 10–15 participants with geographic variation, the Q-GSP accommodates approximately 70 participants. This study aimed to compare the intensive GSP for smoking cessation, as a large group intervention, to the standardized GSP in primary healthcare settings.

## 2. Materials and Methods

This study included 105 participants taking part in the Q-GSP and 14,289 participating in the standardized GSP between 2022 and 2023. Information on the Q-GSP was validated by one municipality in Denmark, with data only available from 2022 to 2023. Only participants who provided consent to a 6-month follow-up were included in the analyses, excluding 1 participant (1%) in the Q-GSP and 703 (5%) in the standardized GSP. Smoking cessation among participants receiving a large group intervention were compared to the standardized GSP. This study included combustible smoking (cigarettes, cigarillos, cigars, and pipes) and smoking was defined as daily smoking. This study is reported according to RECORD guidelines [11]. Data were utilized from the Danish STOPbase for tobacco and nicotine, a comprehensive database containing information on cessation interventions in Denmark, encompassing 95 out of 98 municipalities and covering data from 2006 to 2024 [12]. Smoking cessation interventions in the standardized GSP were offered by local municipalities, and the Q-GSP was offered by the evaluated municipalities. Healthcare professionals often encourage participants to attend smoking cessation interventions locally, and, after admission, participants are free to choose to follow the Q-GSP or the standardized GSP. There is usually a short waiting time, and the timing is agreed upon by the participants and the local clinic. To date, we have not experienced waiting lists or rejections for a smoking cessation intervention. In this study, data on baseline characteristics were collected through a self-reported questionnaire, whereas the 6-week and 6-month data were collected using structured interviews.

### 2.1. The Concept of Q-GSP for Large Groups

Similar to the standardized GSP, the Q-GSP consists of six meetings, each addressing critical aspects of the cessation process. In contrast to the standardized GSP, where a group typically consists of 10–15 participants with a maximum of 20, the Q-GSP includes approximately 70 participants. In this study, the Q-GSP included two interventions: one with 55 participants and the other with 50. The similarities and differences between the Q-GSP and the standardized GSP are presented in Table 1.

Each Q-GSP session begins with a welcome and an overview of the program, followed by an introduction of the topics and guest speakers. Group discussions and sharing are encouraged, and the session concludes with reminders and encouragement. The meetings also include social activities and the opportunity to win prizes for starting the intervention and maintaining the motivation to attend all meetings. Absentees receive follow-up calls for additional support, and the final meeting features a group photo, certificates, and a celebration.

### 2.2. Outcome

The primary outcome of this study was successful smoking cessation defined as complete abstinence from smoking at the end of the 6-week intervention as well as at the 6-month follow-up. Information about the participants’ smoking status was obtained through structured interviews conducted by therapists and coded as a binary variable (yes/no).

### 2.3. Other Variables

Baseline characteristics were collected through self-reported questionnaires at the beginning of the interventions. The characteristics included demographic information in terms of age and sex; a smoking profile, in which a heavy smoker was defined as someone with ≥20 pack-years, a Fagerström score >6, and daily consumption levels of ≥20 cigarettes per day; previous attempts to stop smoking; completed interventions; nicotine replacement therapy; encouragement from healthcare personnel; living with a smoker; living alone; short or long education; and unemployment or employment. Compliance with the intervention was defined as attending at least 75% of the meetings. Satisfaction was measured on a scale of 1 to 5, with high satisfaction defined as a score of 4 or 5. All baseline characteristics were coded as binary variables, except age, which was measured as a continuous variable.

### 2.4. Statistics

The successful smoking cessation rates at the end of the interventions and at the 6-month follow-ups were presented in numbers and percentages. Compliance with the intervention was defined as attending at least 75% of the meetings. Satisfaction was measured on a scale of 1 to 5, with high satisfaction defined as a score of 4 or 5. Relative risk was reported to compare the continuous successful cessation rates at the 6-month follow-up. Missing data were handled using multiple imputation with chained equations [13]. The coefficient of variation between the two interventions was tested post hoc based on the Fagerström score. A significance level of 0.05 was used in all the analyses. R version 4.3.0^®^ was used for all data processing and analyses.

### 2.5. Ethics

All participants registered and provided informed consent using the STOPbase; they could withdraw their consent to the follow-up at any time, without explanation, and with no impact on the intervention. STOPbase was approved by the Danish Data Protection Agency (P-2021-900) and further considered by the Scientific Ethical Committee of the Capital Region (685 27), without further comments.

## 3. Results

In general, there were slightly fewer heavy smokers in the Q-GSP group compared with the standardized GSP group. More participants lived with others, had previously attempted to stop smoking and completed the intervention, and all received nicotine replacement therapy. More were unemployed in this group (Table 2).

The successful cessation rates were significantly higher in the Q-GSP compared to the standardized GSP. At the end of the interventions, the participants in the Q-GSP had a successful cessation rate of 73% compared to 50% in the standardized GSP, and 49% at the 6-month follow-up compared to 31% in the standardized GSP (Table 3). The crude relative risk (RR) was 2.18 (95% CI 1.48 to 3.22), with statistical significance (*p*-value < 0.001). The coefficient of variation between the Q-GSP and standardized GSP based on the Fagerström scores showed low variations (0.43 and 0.41, respectively).

**Figure 1:**
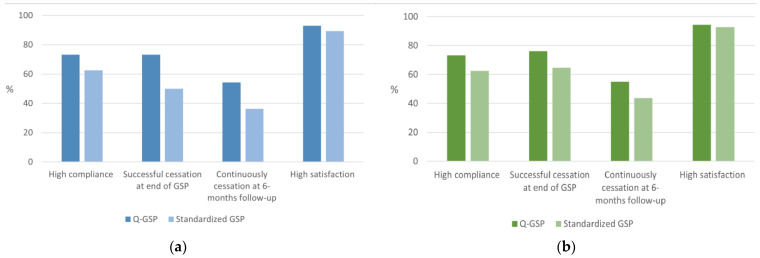
Proportion of indicators for smoking cessation interventions (**a**) for all participants; and (**b**) for participants who complied with ≥75% meeting adherence (per protocol).

All of the key indicators, including compliance, successful cessation rates at the end of the intervention, continuous successful cessation at the 6-month follow-up, and participant satisfaction levels, were higher for the Q-GSP (see Figure 1a for all the participants and Figure 1b for the participants with ≥75% meeting adherence).

## 4. Discussion

The new Q-GSP demonstrated higher successful cessation rates compared to the standardized GSP. At the end of the interventions, almost three out of four participants in the Quitter intervention had successfully stopped smoking, versus half of the population in the standardized GSP. At the 6-month follow-ups, half of the participants in the Q-GSP group and one in three in the standardized GSP group remained smoke-free. The relative risk exhibited a statistically significant twofold difference. Similar, but higher, cessation rates were found for the participants with a high compliance, where the difference in percentage points was lower but still significant. Both interventions led to high satisfaction levels overall.

The participants in the Q-GSP group were generally better positioned for successful cessation based on their baseline characteristics and known predictors for smoking cessation, which may have contributed to the differences among the groups. Further, the Q-GSP includes social activities, entertainment, and the opportunity to win prizes as part of the program to encourage the participants to start the intervention and attend all the meetings. The participants’ preference for the structure of the intervention may have influenced their participation and the effect rates across the two interventions. Previous results in the literature indicate that multiple factors related to smoking behavior and nicotine dependence, cognitive factors, motivation to stop smoking, and social context play crucial roles in participants’ recruitment, attendance, and cessation rates [1,2,3]. Despite the significant differences in successful cessation, the results must be interpreted with caution due to differences such as fewer heavy smokers, a lower nicotine dependency, a higher use of nicotine replacement therapy, and the completion rate among the participants in the Q-GSP. This may partially explain the better outcomes in the Q-GSP interventions. Moreover, a high proportion of unemployment may reduce cessation success. Interestingly, the coefficient of variance in the Fagerström scores was low (Table 2).

### Group Intervention

A systematic review and meta-analysis of 19 randomized controlled trials (RCTs) demonstrated a threefold higher odds of 6-month continuous abstinence from smoking in a group-based intervention compared to the usual individual-based treatments [3]. Similar results were presented in a Cochrane review from 2017 [1]. This is a notable and important consideration given the potential cost-effectiveness of group-based versus individual counseling. The Quitter intervention’s higher cessation rates highlight the effectiveness of large group-based smoking cessation interventions. These findings suggest that large group-based I-SCIs are equally effective in achieving successful smoking cessation.

Group programs offer participants a unique opportunity for social learning, such as sharing skills and knowledge and receiving support from fellow group members [1,2,3]. An older study examining a smoking cessation program delivered in a large group format, with approximately 100 participants attending eight 90-min sessions over four months, reported cessation rates of 39%, 32%, and 26% at 3, 6, and 12 months, respectively [14]. The 6-month cessation rate was lower compared to the Quitter intervention but almost similar to the standardized I-SCI in Denmark. All interventions included patient education on the effects of smoking, the establishment of a group cessation date, and behavioral modification strategies. However, both the Quitter intervention and the standardized I-SCI in Denmark were based on the GSP and were more intensively structured than that employed in the study conducted by Carlson et al. Additionally, the differences in program format and intensity, as well as the years between the studies, may account for the variance in the 6-month cessation rates [14].

Group support emerges as a significant factor for smoking cessation, with participants forming connections and discussing their progress even within large cohorts. Notably, the most frequently cited beneficial aspect of the program in the study by Carlson et al. was the supportive nature of the groups. Despite average group sizes of approximately 100 individuals, the majority of the participants reported feeling supported [14]. These findings suggest that employing a flexible and efficient cognitive behavioral approach to smoking cessation can be effective even with large groups of participants.

Overall, a significant number of smokers are motivated to stop smoking and make frequent cessation attempts [15,16]. Additionally, a lack of awareness hinders successful smoking cessation, even among those who are determined to become smoke-free.

Recruiting participants for smoking cessation is challenging, with low participation rates [17]. A systematic review and meta-regression analysis aimed to identify the predictors affecting participant eligibility, recruitment rates, and retention rates in smoking cessation RCTs. This study found that indirect recruitment methods, e.g., posters or websites, led to lower rates compared to direct contact methods such as contact from healthcare providers. Self-help interventions demonstrated lower abstinence rates than in-person interventions, while financial incentives improved retention. Many anticipated predictors of trial participation, such as nicotine dependence and age, did not show significant associations [17]. These findings are consistent with previous research reporting that heavy smoking and high nicotine dependence were common among the participants of a smoking cessation intervention and were related to cessation attempts among adolescents [17]. Additionally, reactive recruitment methods, where smokers must initiate contact after being advertised an intervention, mostly attract motivated individuals to stop smoking, limiting generalizability. In contrast, proactive recruitment, where individuals are directly contacted and offered services, obtains a more representative population [15]. Studies have shown that direct contact by healthcare professionals effectively targets eligible patients for smoking cessation studies [18,19,20]. In the UK, proactive recruitment using computerized patient records to identify smokers is a crucial method, and improving clinical data recording can enhance recruitment and overall patient care [15].

The cost-effectiveness and social learning opportunities inherent in group programs offer significant public health benefits, as they provide robust support networks and shared learning experiences [1,3,14]. Given the challenges in recruiting participants for smoking cessation programs, indirect methods instead of proactive and direct recruitment strategies, as well as understanding the predictors of participation, are essential for optimizing the reach and impact of these interventions [17].

This study has several strengths, including comprehensive and validated data obtained from the Danish STOPbase for tobacco and nicotine, detailed and well-characterized interventions, and a comparative analysis between the large group-based Quitter intervention and the standardized I-SCI in Denmark. However, the limitations include a reliance on self-reported data; the inclusion of participants from a single municipality, which potentially introduces selection bias; the inability to adjust for potential confounders; and limited generalizability. Self-reported successful cessation may be overestimated, but is seldom underestimated. However, collecting data using interviews is more reliable compared to participants filling in questionnaires separately by themselves [21]. As the participants in the Q-GSP were better positioned for a successful cessation, the differences in the results were limited by potential confounders. The compatibility of the results across the groups may have been improved if the participants were matched on potential confounders to the standardized intervention. However, the post hoc coefficient of variance in the Fagerström scores showed a low variation. The results may have been impacted by periodic differences in the delivery of the 6-week interventions in the standardized GSP group. Only the information from the 6-month follow-up was available; therefore, results related to a periodical process, and a longer follow-up would be relevant in future studies. Large group interventions for smoking cessation need to be tested in sizeable studies. Future studies should explore this intervention in different demographic and geographic contexts to determine its broader generalizability. Furthermore, for future studies using very large databases, matching the participants in the design of the study might improve the compatibility of the results across groups. It might also be helpful for future studies to include an evaluation for patients to grade the most important changes.

## 5. Conclusions

In conclusion, this study demonstrates that large group-based smoking cessation interventions may appear to be at least as effective as the standardized I-SCI in Denmark. The findings indicate higher rates of successful cessation and underscore the potential of these programs to be implemented as strategies aimed at reducing smoking prevalence. This study could be a steppingstone for further research.

## Figures and Tables

**Table 1:** The standardized and Quitter Golden Standard Programs.

Standardized Golden Standard Program	Quitter Golden Standard Program
Free of charge	Free of charge
5–6 meetings in 6 weeks	6 meetings in 6 weeks
No contact with participants who fail to attend	Phone call to participants who fail to attend the first meeting
Relapse prevention is recommended 3 months after the quit date	Relapse prevention is recommended 3 months after the quit date
Group or individual sessions	Large group sessions of approximately 70 and smaller groups of approximately 10 for counseling
Structured manual-based patient education	Structured manual-based patient education
Trained therapist	Trained therapist
1–2 therapists per session	7 therapists at each session
Self-referral or from healthcare personnel	Self-referral or from healthcare personnel
Nicotine replacement therapy is part of the patient education, offered free of charge on an individual level based on participants’ Fagerström score and personal preferences	Nicotine replacement therapy is part of the patient education, offered free of charge on an individual level based on participants’ Fagerström score and personal preferences
Information and education about smoking and smoking cessation interventions	Information and education about smoking and smoking cessation interventions
Follow-up at six months	Follow-up at six months
No contact with participants who discontinue the program during the 6-week intervention	Contact with participants who discontinue the program during the 6-week intervention
Re-intervention offered at 6-month follow-up for those who still smoke	Re-intervention offered at 6-month follow-up for those who still smoke
An average of 6 participants	Approximately 70 participants
None	Prize raffle at each meeting
None	Activities in a social context such as performance of a choir

**Table 2:** Characteristics of the Quitter and standardized Golden Standard Programs at baseline and for the successful quitters after 6 months.

		Q-GSP ^1^ n (%)	6-Month Cessation n (%)	Standardized GSP ^2^ n (%)	6-Month Cessation n (%)
Total, n		105	54	14,289	4155
Age, median (range)		56 (47–65)		55 (41–64)	
Male		49 (46.7)	23 (42.5)	6867 (48.1)	2120 (51.0)
**Smoking profile**					
Heavy smoker ^3^		74 (70.5)	36 (66.6)	10,872 (76.1)	3017 (72.6)
	≥20 pack-years	66 (64.7)	33 (61.1)	9422 (68.1)	2738 (65.9)
	Fagerström 7–10 points	19 (18.8)	4 (7.4)	4359 (31.4)	1193 (28.7)
	≥20 cigarettes per day	14 (20.0)	7 (12.9)	2807 (30.5)	1064 (25.6)
Previous attempts to stop smoking		68 (64.8)	33 (61.1)	8168 (58.4)	2518 (60.6)
Completed intervention		77 (73.3)	40 (74.1)	8939 (62.6)	3224 (77.6)
Nicotine replacement therapy		105 (100.0)	54 (100.0)	8799 (61.6)	2709 (65.2)
Encouraged by healthcare personnel		72 (93.5)	51 (94.4)	9727 (95.6)	3885 (93.5)
Live with a smoker		27 (25.7)	14 (25.9)	3761 (26.8)	1072 (25.8)
Live alone		36 (34.6)	20 (37.1)	6134 (43.8)	1641 (39.5)
Short education		64 (65.3)	35 (64.8)	9045 (67.9)	2717 (65.4)
Unemployment		60 (57.7)	29 (53.7)	5370 (39.4)	1849 (44.5)

^1^ Quitter Golden Standard Program; ^2^ Golden Standard Program; ^3^ Heavy smoker: based on ≥20 pack-years, Fagerström scores > 6, and daily consumption levels of ≥20 cigarettes per day.

**Table 3:** Smoking cessation rates using the Quitter and standardized Golden Standard Programs.

	Q-GSP ^1^, n (%; 95%CI)	Standardized GSP ^2^, n (%; 95%CI)
Number of participants	104	13,586
Successful cessation at end of intervention	76 (73.2) (63.6–81.0)	6836 (50.0) (49.2–50.9)
Continuous successful cessation at 6-month follow-up	51 (49.0) (39.6–58.5)	4155 (30.6) (29.8–31.4)

^1^ Quitter Golden Standard Program; ^2^ Golden Standard Program.

## Data Availability

The anonymized data and statistical codes are available from the corresponding author upon reasonable request.
